# Supplementary material for: Metformin Treatment Induces Different Response in Pheochromocytoma/Paraganglioma Tumour Cells and in Primary Fibroblasts
Source: Cancers (Basel). 2022 Jul 17;14(14):3471. doi: 10.3390/cancers14143471 (PMC9320533; doi:10.3390/cancers14143471)
Supplement: Supplementary file 1 [file cancers-14-03471-s001.zip › cancers-1828021-Supplementary Material Figure S1.pdf]

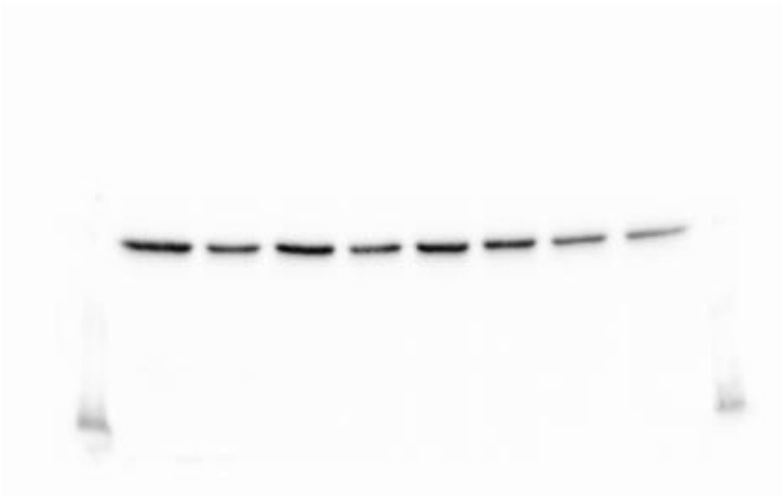

Cyclin D1, Figure 1

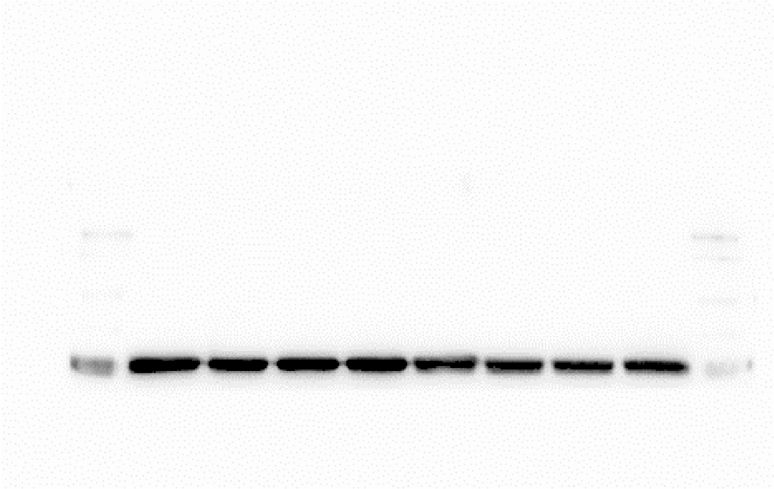

α Tubulin, Fig. 1

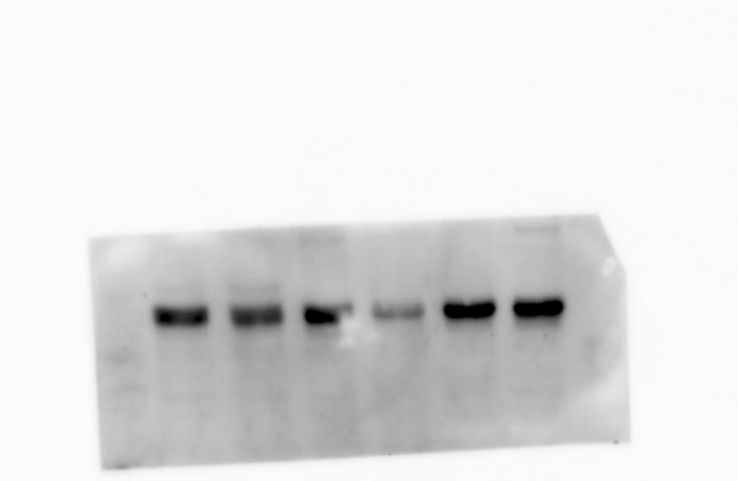

p-Akt, Figure 7

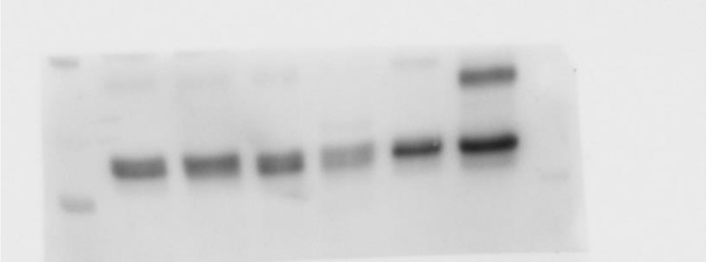

Akt, Figure 7

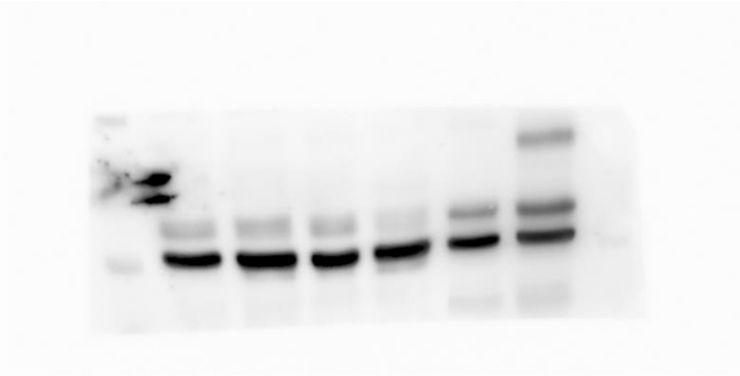

α tubulin, Figure 7

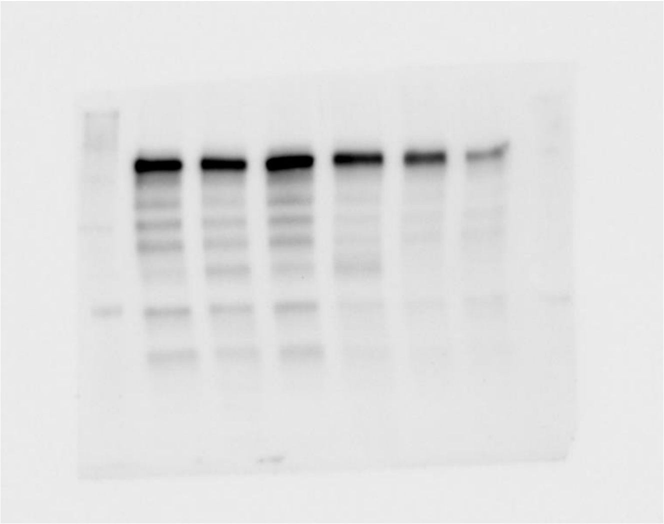

N-Cadherin, Figure 7

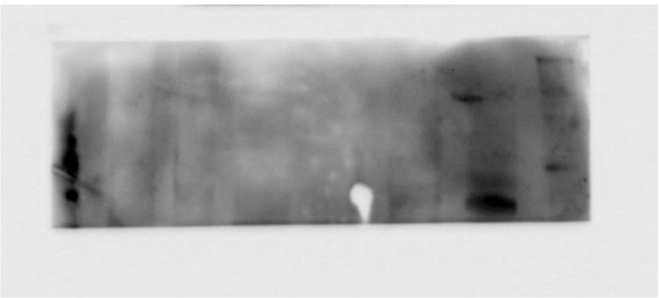

E-Cadherin, Figure 7

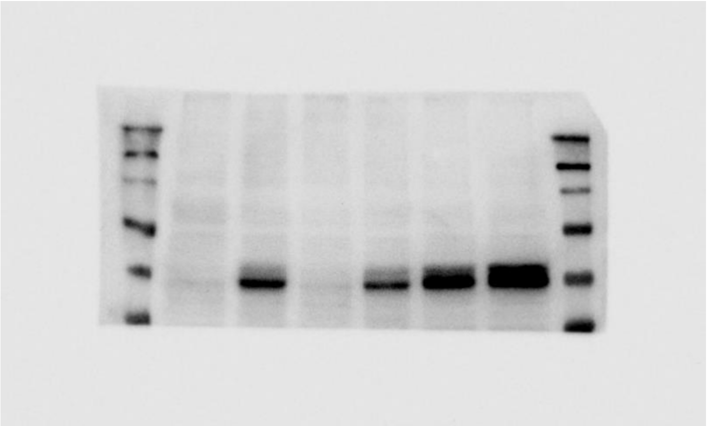

p-AMPK, Figure 7

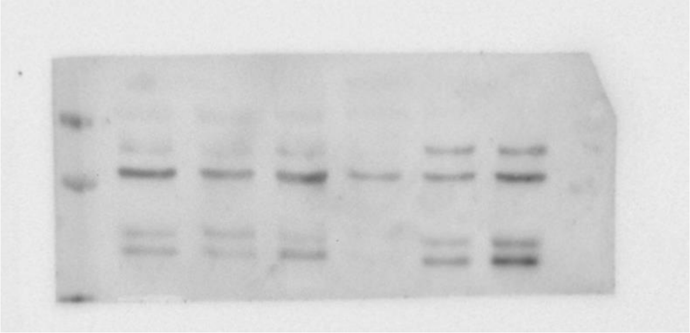

P-Erk1/2, Figure 7

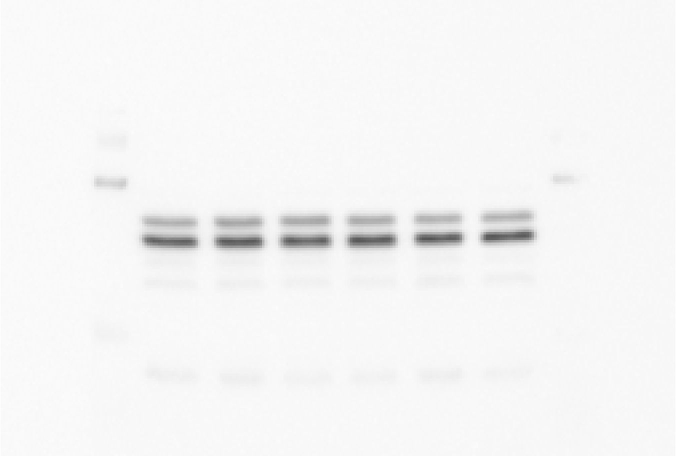

Erk1/2, Figure 7
